# The Influence of Object Relative Size on Priming and Explicit Memory

**DOI:** 10.1371/journal.pone.0003109

**Published:** 2008-09-01

**Authors:** Bob Uttl, Peter Graf, Amy L. Siegenthaler

**Affiliations:** 1 Red Deer College, Red Deer, Alberta, Canada; 2 Department of Psychology, University of British Columbia, Vancouver, British Columbia, Canada; 3 Lifemark Health Centre, Calgary, Alberta, Canada; University of Minnesota, United States of America

## Abstract

We investigated the effects of object relative size on priming and explicit memory for color photos of common objects. Participants were presented with color photos of pairs of objects displayed in either appropriate or inappropriate relative sizes. Implicit memory was assessed by speed of object size ratings whereas explicit memory was assessed by an old/new recognition test. Study-to-test changes in relative size reduced both priming and explicit memory and had large effects for objects displayed in large vs. small size at test. Our findings of substantial size-specific influences on priming with common objects under some but not other conditions are consistent with instance views of object perception and priming but inconsistent with structural description views.

## Introduction

To what extent does the size of objects influence our ability to identify and remember them? Intuitively, size would seem to be an important object attribute since we often rely upon knowledge of object size for correct identification; for example, we learn to distinguish between a viola and a violin primarily by their relative size. The majority of recent experimental work, however, has suggested that object size may not be represented as a fundamental attribute of object representations used for object identification (see Table 1); for example, Uttl, Graf, andSiegenthaler [1] found that changes in the *absolute* size of objects between study and test had very little influence on priming. It may be, however, that the typical way in which participants' knowledge of object size is examined in the laboratory (e.g., on computer screens or paper cards) does not sufficiently tax their knowledge of object size; for example, we are all used to seeing objects of incredibly diverse sizes depicted on computer and television screens, and these do not look odd to us. When objects are presented in unfamiliar *relative* sizes, however, they do tend to look odd, and attract our attention; we notice immediately that something is wrong when the children appear much smaller than the plants as in the movie *Honey*, *I Shrunk the Kids!* By this line of reasoning, one would expect that when objects' relative size violates pre-existing knowledge, it can become part of episodic memory representations, and thereby influence the magnitude of priming. However, no study to date has investigated the influence of object relative size on implicit and explicit memory.

**Table 1:** Previous studies of object size effects on priming.

Source	Exp.	Test task	Size change	Stimuli	Effect	Comments
Biederman & Cooper [31]	1	naming	3.5–6.2°	LD	+	NExp. prior
Biederman & Cooper [31]	3	naming	3.5–6.2°	LD	+	NExp. prior
Cave & Squire [49]	2	naming	1∶1.5	LD	+	
Fiser & Biederman [50]	1	naming	3.5–6.2°	PH (b&w)	+	NExp. prior
Zimmer [51]	1	size judgments	1∶2.1	LD	n/i	no priming
Zimmer [51]	2	size judgments	1∶2.1	LD	n/i	no priming
Zimmer [51]	3	word-picture matching	5–8 cm	LD	−	
Srinivas [30]	1	size typicality	8.8–17.22°	LD	++	
Srinivas [30]	2	fragment identification	8.8–17.22°	LD	++	
Seamon et al. [52]	2	affective preference	1∶2.5	LD	−	MExp
Furmanski & Engel [53]	3	identification thresholds	8.2–16.5°	PH (b&w)	+	MExp/Transfer
Furmanski & Engel [53]	4	identification threshold	8.2–16.5°	PH (b&w)	+	Mexp/Transfer
Stankiewicz & Hummel [54]	2	naming	2.5–5°	LD	+	
Ryan et al. [55]	1	naming	5.7–10°	LD	−	
Uttl et al. [1]	1	identification threshold	4–16°	PH (color)	+	

*Note.* LD = line drawings; PH = photographs; MExp = participants were given multiple exposures to objects during the study; MExp/Transfer = hundreds of study trials with each object followed by transfer block trials; NExp prior = participants were asked to read names of all objects prior to the experiment; − = nonsignificant size difference in opposite direction; + = nonsignificant but numerically larger size effect; ++ = significant size effect; n/i = not interpretable.

Whether non-structural object attributes such as size, color, and orientation have an effect on identification and priming has important implications for theories of object identification and priming. For example, according to Biederman's [2] recognition-by-components model of object identification, neither geons nor the relations among them are associated with non-structural information such as orientation, color, and size information, and thus, any influence due to these non-structural attributes on identification can only be due to lower level processes, such as edge and shape detectors [2], [3]. Since each study encounter with an object is thought to prime only a structural model, no influence of non-structural attributes ought to be observed on priming. Recent work from our lab and others, however, contradict the predictions of the structural description account. For example, we have shown that repetition priming can be influenced by study-test changes in non-structural object attributes such as object orientation [4] and color [5]. Similarly, working with novel line shapes and computer-generated novel objects, Tarr and his colleagues have found that study-test format specific effects on repetition priming depend on participants' familiarity with particular views [6], [7].

Findings such as these are more in line with models of repetition priming which contend that repetition priming is hyper-specific to study-test changes in stimulus format [8]–[10], than with structural description accounts of object identification. These claims of hyper-specificity of priming to sensory and perceptual attributes of stimuli arose from research on repetition priming with words showing substantial effects due to study-test manipulations of font size and type [11]–[15], display orientation [11], [16], presentation modality [10], [17], [18], or presentation voice [19]–[21]. Contradicting the claims of priming hyperspecificity, however, recently some authors have emphasized the variability of the findings obtained with words (e.g.,[11]) and have noted that study-test changes in stimulus format sometimes influences and sometimes does not influence performance on repetition priming or explicit memory tests. As a result, more flexible instance-based accounts of repetition priming have been proposed (e.g., [11], [22]–[25]). Indeed, the word priming literature seems to have abandoned accounts relying only on structural or abstract representations in favor of either instance-based accounts or hybrid accounts that postulate both instances and abstract representations to explain the variability of priming effects (e.g., [25], [26]).

Similarly, Uttl and Graf [4] examined the effect of study to test changes in object orientation on identification and priming for objects that are seen in predominantly a *cardinal orientation* (e.g., a helicopter) vs. objects that are seen in a variety of orientations and have no cardinal orientation (e.g., scissors). Whereas study to test changes in object orientation had no effect on priming for non-cardinal objects, they had a large influence on priming for cardinal objects but only when cardinal objects were shown in a non-cardinal orientation at test. Similar to words, neither structural description models (e.g., [2], [27]) nor models that postulate that priming is hyperspecific to study to test changes in stimulus format can explain these findings (e.g., [8]–[10]). However, Uttl and Graf's findings are easily explained by any instance view and some hybrid views of priming (e.g., [11], [22]–[26], [28], [29]).

Prior research examining the effects of object size on the magnitude of priming is summarized in Table 1. Previous studies have employed a variety of testing procedures including naming, picture-fragment completion, and identification thresholds, but have used primarily line-drawing stimuli, and only 1∶2 study-test size changes (but see [1]). Although the majority of studies have revealed numerically larger priming effects for objects displayed in the same size at study and test, they have rarely found such effects to be statistically significant (but see [30], Experiments 1 & 2). To ensure that such findings are not due to low power or other experimental confounds (e.g., multiple item exposures), our previous study [1] was designed to be powerful enough to detect a 20% reduction in priming (e.g., large object set, large range of object sizes, color photographs of objects), yet still found only a minimal, statistically marginal effect of study-test changes in size on priming. As suggested, earlier, however, this outcome may only mean that the size manipulation was too small in that all objects were represented as smaller than a standard computer screen, whereas our real-life experience entails perceiving objects much smaller than to much larger than our physical body size. Furthermore, instance views of priming suggest that the effects of study-test changes in object size on priming may depend on participants' familiarity with a specific object size; specifically, priming will be larger for objects tested in an unfamiliar size when they were studied in an unfamiliar than when studied in a familiar size. Given that modern undergraduates are presumably experts at recognizing all variety of object sizes on video screens and given further the unwieldiness of bringing in a sufficient number of real objects in various familiar and unfamiliar sizes to the standard psychology laboratory, we chose to investigate this idea by manipulating the relative size in which objects are presented on the computer screen. We hypothesized that when the size of an object displayed in context violates our expectations (e.g., a large book next to a small bus), larger priming effects may emerge due to prior study encounters with the object displayed in an unfamiliar versus a familiar relative size.

In contrast to priming, previous research on object size has shown that it does have an effect on identification and on explicit memory (the ability to recognize them as having been encountered previously; see e.g., [1], [31], [32]; see Table 2). Moreover, consistent with previous findings with human faces [33], [34], Uttl et al. [1] recently reported that the effects of changing object size between study and test on explicit memory are asymmetric. Specifically, performance on an old/new recognition explicit memory test was affected by study to test changes in object size when test objects were large but not when test objects were small.

**Table 2:** Previous studies of object size effects on old/new recognition.

Source	Exp.	Size change	Stimuli	Effect	Comments
Biederman & Cooper [31]	2	3.5–6.2°	LD	++	
Jolicoeur [32]	1		LD	++	
Srinivas [30]	3	8.8–17.22°	LD	++	CEs
Seamon et al. [52]	2	1∶2.5	LD	++	MExp
Zimmer [51]	1	1∶2.1	LD	n/i	CEs
Zimmer [51]	2	1∶2.1	LD	++	CEs
Zimmer [51]	3	1∶1.6	LD	n/i	CEs
Uttl et al. [1]	1	4–16°	PH (color)	++	for large at test only

*Note.* LD = line drawings; PH = photographs; MExp = participants were given multiple exposures to objects during the study; MExp/TP = many identification trials followed by transfer block trials; NExp prior = participants were asked to read names of all objects prior to the experiment; − = nonsignificant size difference in opposite direction; + = nonsignificant but numerically larger size effect; ++ = significant size effect; n/i = results are not interpretable; CEs = ceiling effects.

This asymmetry in old/new recognition performance is easily explained by the well-known limitations of the human visual system, including its increasingly poor processing of higher spatial frequencies due to the acuity limitations related to spatial density and distribution of rods and cones on human retina [35]–[37]. Specifically, encountering small objects at study will result in less detailed representations than encountering large objects as the human visual system processes higher spatial frequencies (detail) increasingly more poorly. The extra detail included in the study representation of large objects will not be beneficial on a subsequent test when the test object is small, however, and therefore, lacks detailed information. In contrast, the extra detail included in the study representation of large objects is helpful when the test object is also large, and therefore, includes detailed information [1], [34].

One may ask why this asymmetric old/new recognition effect reported by Uttl et al. [1] (see also [33] for similar asymmetric effects found with photographs of faces) has not been found in previous studies of object size effects on old/new recognition listed in Table 2. A simple explanation for this discrepancy is that in contrast to Uttl et al. [1], previous studies of object size effects have used primarily line-drawings that scale with no loss of detail and therefore establish equally rich memory representations. Accordingly, the results of the present study should replicate these asymmetric old/new recognition effects reported by Uttl et al. [1] and also find asymmetric priming effects, provided that relative size has an effect on priming.

In addition to examining whether the relative size of objects influences priming and explicit memory, we also hoped to rectify some possible shortcomings of earlier research on object identification and priming. To this end, we used color photographs of common objects rather than line-drawings, employed a one-week study-test delay to limit ceiling effects on explicit memory test performance, used a large number of objects to ensure the generalizability of our findings, and designed the study to be powerful enough to detect a 33% reduction in priming due to object size manipulations. Objects were presented by means of a fade-in procedure in which target-relevant information accumulates over time during both study and test (see [1], [4], [5]). This procedure mimics the perceptual experience of identifying objects through a lifting fog, and is similar to picture-fragment completion methods used by prior investigations (e.g., [30], [38]) but has greater measurement precision and high reliability.

Participants were presented with photos of two common objects on each trial; one object was designated the context and the other object was designed the target. Contexts and targets were selected so as to be approximately the same size in real life. To manipulate the object's relative size, the displays showed the target either in an appropriate relative size (i.e., the same size as the context) or in an inappropriate relative size (i.e., much smaller or much larger than the context). To emphasize the encoding of relative size information, the study task focused participants' attention on object size by requiring them to decide which object was more likely to be larger in real life. Participants studied each context-target pair twice: in the first study phase, both objects were slowly faded in until participants made a relative size decision; in the second study phase, the context object appeared first fully faded-in and the target object was slowly faded in until participants made the relative size decision. One week later, implicit memory was assessed by means of the same rating task and presentation method as the second study phase; priming was indexed by the facilitation in the rating speed of studied versus non-studied targets. Explicit memory was tested by means of an old/new recognition test. On each trial, the context object was displayed first, and participants were required to classify it as either artificial or natural ensuring that participants paid attention to the context object. Next, the target object was slowly faded in until participants made an old/new decision. For both implicit and explicit tasks, each target was displayed in either the same relative size at study and test or in different relative sizes at study and test. Both implicit and explicit memory tests were given after a 1-week delay to avoid ceiling effects on the old/new recognition explicit memory test.

A secondary question addressed by the study was whether the influence of the relative size manipulation on priming and explicit memory could be mediated by specific associations between the objects in each context-target pair or by relative size per se. For this purpose, each target appeared either with the same context object at study and test or with a different context object at study and test. Previous research has shown that context-target associations can influence both the magnitude of priming on word stem completion tests and performance on verbal explicit memory tests such as cued recall tests (e.g., [39]–[43]). For example, Graf and Schacter [41] found associative priming, larger effects for targets presented with the same versus different contexts, on a word stem completion test, and they showed that performance on a cued recall test is higher when targets appear with the same contexts versus different contexts at study and test. Consistent with these findings, one might expect associative effects on priming and on old/new recognition test performance in the present experiment.

## Methods

### Participants and design

**Figure 1:**
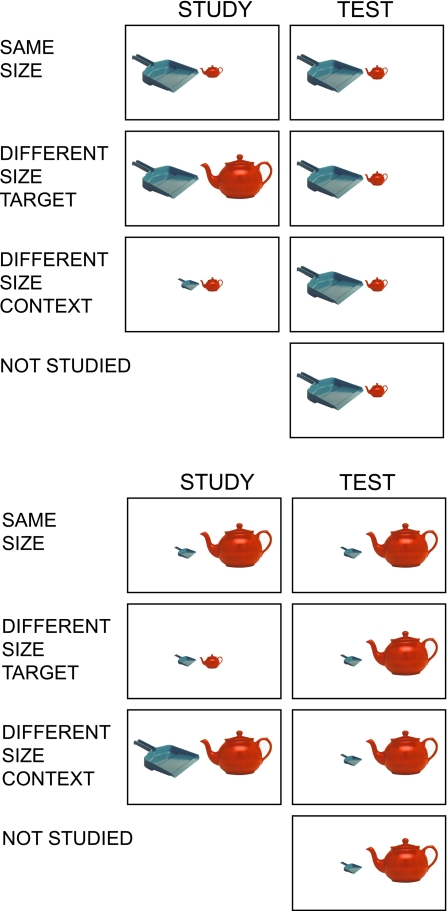
Examples of object pairs displayed in large vs. small relative size at test by various study conditions.

One hundred sixty undergraduate students participated for course credit. The design had one between-subjects factor: test type (implicit, explicit) and four within-subjects factors: history (studied, non-studied), target size at test (small, large), study/test relative size (same size, different size target, different size context), and study/test context (intact, recombined). Ninety-six participants were assigned to the implicit memory test and 64 were assigned to the explicit memory test condition. Figure 1 shows the critical conditions and how object photos appeared in each of them (the figure shows critical conditions only for the intact study/test context condition).

### Materials

A set of 360 color photos of common objects was obtained and digitized in true color mode. Each object was ‘cut out’ from its background, superimposed and centered on a 1280 by 960 pixels white background, and then scaled to fill two different rectangles, either 512 by 384 pixels for large displays or 160 by 120 pixels for small displays.

Ninety-six critical pairs (i.e., context-target pairs) were created by repeating the following procedure. First, four photos of objects were selected randomly, without replacement, from the pool of available photos, subject to the constraint that they had to be all about equally large in real-life. Second, two of these four objects were randomly chosen as targets, leaving the other two objects as contexts. Third, the targets were randomly combined with the contexts to create two *intact pairs*. *Recombined pairs* were formed by switching the context objects between the two intact pairs. Finally, all pairs were checked, and if any pair, either intact or recombined, had two strongly-associated objects (e.g., a teacup and a teaspoon), the subset of objects was returned to the pool of items. This procedure was followed until all pairs met the criteria.

For purposes of counterbalancing items across participants and conditions, critical context-target pairs were divided randomly into 16 sets (Cr_1_ to Cr_16_). Each target from any of these sets was associated with two contexts in the same set. Each target was associated with one context in the intact pair and with another context in the recombined pair. This materials arrangement ensured that both of the contexts associated with each target also occurred in the same set of context-target pairs.

Seven practice pairs (Pr) and 48 filler pairs (Fi) were formed in exactly the same way as the critical pairs. The former pairs were used for instruction and practice and the latter pairs were used to equate the number of studied and non-studied pairs on the old/new recognition test. Finally, sixteen *catch pairs* (Ca) were formed by combining two unrelated objects in such a way that, in real life, each target object was either much smaller or much larger than its assigned context object. The catch pairs were employed to ensure that participants followed the relative size rating instructions and were not simply responding based on target object display size.

All picture pairs were displayed on a 17-inch color monitor, driven by a color graphics card operating in 16.7 million color mode at a resolution of 1024 by 768 pixels.

The study was approved by the Research Ethics Committee, Department of Psychology, University of British Columbia, BC, Canada, and a written consent was obtained from each participant prior to participation in the study.

### Procedure

Participants participated in two sessions, a study and a test session, separated by one week. Each session lasted between 45 and 60 minutes. The experiment was described as examining perception of pictures of objects. The study session had two phases both requiring participants to decide which one of the two objects was more likely to be larger in real-life. At the beginning of the first phase, participants were presented with three practice and two catch context-target pairs that were color printed on 11 by 14 inch paper cards. Using these cards, the experimenter explained the size decision task. For each pair of objects, participants were required to decide which one was likely to be larger in real-life. They practiced this task until the experimenter was satisfied that they understood the task.

Following instructions and practice, participants performed the relative size decision test administered on a computer. On each trial, a context object was displayed either on the left or on the right of the computer screen with the target object displayed on the other side of the screen. Both objects were faded-in simultaneously. The fade-in procedure operated on the 1024 by 768 pixel map of each picture. The program stepped through this map, in a pseudo-random sequence, and on each step, it turned on one of the not-yet-turned-on pixels. The generator of a pseudo-random sequence was designed in such a way that no pixel in the map was visited twice (i.e., sampling without replacement), and the relations between the number of pixels turned on and the time elapsed from the start of fade-in was linear (20 s were required to turn on all pixels). The number key press generated a software interrupt, stopped the fade-in procedure, and caused the current fade-in level to be recorded in terms of the proportion of pixels turned on prior to the keyboard button press. Participants were instructed “to decide which one of the two objects, in your opinion, is more likely to be larger in real-life” and were instructed to make each decision as quickly and accurately as possible. They pressed 1 on the keyboard number pad if the object on the left side was larger, and pressed 2 if the object on the right was larger.

During the second phase, participants performed the same relative size decision test with the same context-target pairs, with the following modification: on each trial, one object (the context object) appeared immediately; after a brief delay (1.5 seconds) a second object (the target object) was slowly faded in until participants made the relative size decision by pressing the appropriate number key. Following instructions and practice, participants performed the fade-in relative size decision test on exactly the same context-target pairs (i.e., the same pairs presented in the same relative and absolute sizes) as in phase 1 except that context-target pairs were presented in a new random sequence. The Phase 1 and Phase 2 pairs (the study list items) included 6 sets of intact pairs (Cr_1_, Cr_2_, Cr_3_, Cr_5_, Cr_6_, Cr_7_) and 6 sets of recombined pairs (Cr_9_, Cr_10_, Cr_11_, Cr_13_, Cr_14_, Cr_15_). In addition, the Phase 1 and Phase 2 pairs also included four sets of catch pairs (Ca_1_ to Ca_4_), one set in each of the context-target size combinations, to ensure that participants followed the size decision instructions.

The second session (Phase 3) followed one week later; at this time, participants were given either the implicit or explicit memory test. The implicit memory test was the object size decision task used during study. The explicit memory test was an old/new recognition test. On each trial of the old/new recognition test, the context and target were presented in exactly the same way as they were presented during the implicit memory test with the following exceptions: (1) when the context appeared, participants were required to say aloud whether the object was artificial or natural, to ensure that they attended to the context object; and (2) their task was to decide, as quickly and accurately as possible, whether each target (the faded-in object) had appeared previously during the study session, regardless of the size and regardless of the context in which it had appeared. Participants pressed ‘1’ on the keyboard number pad if the target was old, and they pressed ‘2’ if the target was new. The computer recorded both the accuracy and speed of each decision. Immediately after each key press, both the context and target were erased, and the next trial began in about two seconds.

The Phase 3 pairs included 6 sets of pairs that were previously studied intact (intact pair sets: Cr_1_, Cr_2_, Cr_3_, Cr_5_, Cr_6_, Cr_7_), 6 sets of pairs that were previously studied recombined (recombined pair sets: Cr_9_, Cr_10_, Cr_11_, Cr_13_, Cr_14_, Cr_15_), and 4 sets of pairs that were not studied (Cr_4_, Cr_8_, Cr_12_, Cr_16_). Moreover, as shown in Figure 1, the previously studied target objects could appear in the same size with the same size context (e.g., Cr_1_, Cr_5_), in a different size with the same size context (e.g., Cr_2_, Cr_6_) or in the same size with a different size context (e.g., Cr_3_, Cr_7_). Phase 3 pairs also included two sets of filler pairs to equalize the number of studied versus non-studied targets and contexts and eight sets of catch pairs (four of them previously-studied [intact pair sets Ca_1_ to Ca_4_] and 4 of them non-studied [intact pair sets Ca_5_ to Ca_8_]) to ensure that participants followed the object size decision instructions appropriately. Table 3 shows an example of how sets of context-target pairs were assigned for one participant.

**Table 3:** An example of how sets of context-target pairs were assigned for one participant.

Phase 1 & 2 pairs (72Cr+8Ca)	Phase 3 pairs (96Cr+48Fi+16Ca)	Pair set	# of pairs
I-C_L_T_S_	I-C_L_T_S_ (S)	Cr_1_	6
I-C_L_T_L_	I-C_L_T_S_ (D/T)	Cr_2_	6
I-C_S_T_S_	I-C_L_T_S_ (D/C)	Cr_3_	6
	I-C_L_T_S_ (N)	Cr_4_	6
R-C_L_T_S_	I-C_L_T_S_ (S)	Cr_5_	6
R-C_L_T_L_	I-C_L_T_S_ (D/T)	Cr_6_	6
R-C_S_T_S_	I-C_L_T_S_ (D/C)	Cr_7_	6
	I-C_L_T_S_ (N)	Cr_8_	6
I-C_S_T_L_	I-C_S_T_L_ (S)	Cr_9_	6
I-C_S_T_S_	I-C_S_T_L_ (D/T)	Cr_10_	6
I-C_L_T_L_	I-C_S_T_L_ (D/C)	Cr_11_	6
	I-C_S_T_L_ (N)	Cr_12_	6
R-C_S_T_L_	I-C_S_T_L_ (S)	Cr_13_	6
R-C_S_T_S_	I-C_S_T_L_ (D/T)	Cr_14_	6
R-C_L_T_L_	I-C_S_T_L_ (D/C)	Cr_15_	6
	I-C_S_T_L_ (N)	Cr_16_	6
	I-C_L_T_S_ (N)	Fi_1_	24
	I-C_S_T_L_ (N)	Fi_2_	24
R-C_L_T_S_	I-C_L_T_S_ (S)	Ca_1_	2
R-C_L_T_L_	I-C_L_T_L_ (S)	Ca_2_	2
R-C_S_T_L_	I-C_S_T_L_ (S)	Ca_3_	2
R-C_S_T_S_	I-C_S_T_S_ (S)	Ca_4_	2
	I-C_L_T_S_ (N)	Ca_5_	2
	I-C_L_T_L_ (N)	Ca_6_	2
	I-C_S_T_L_ (N)	Ca_7_	2
	I-C_S_T_S_ (N)	Ca_8_	2

*Note*. In this table, intact pairs are preceded by ‘I’ and re-arranged pairs by ‘R’. Contexts are denoted by ‘C’ and targets are denoted by ‘T’. Contexts or targets displayed in small size have subscript ‘S’ and contexts and targets displayed in large size have subscripts ‘L’. The letters in parentheses indicate whether the target was displayed in the same size condition (S), in a different size target condition (D/T), in a different size context condition (D/C), or whether it was not studied (N). Cr = critical pairs, Fi = fixed pairs, Ca = catch pairs.

Across participants, counterbalancing ensured that each set of critical pairs (Cr_1_ to Cr_16_) appeared equally often in each of the 16 experimental conditions. Finally, context-target pairs were presented in a random order in each phase, and the order was randomized for each participant.

## Results

The critical dependent measure on the object decision test was the proportion of pixels required for making decisions for studied and non-studied targets in each experimental condition. For the old/new recognition test, the dependent measures were hits (the proportion of targets correctly classified as old/studied) and correct rejections (the proportion of targets correctly classified as new/non-studied). The alpha was set at .05 for all statistical tests.

### Object size decisions and priming

**Figure 2:**
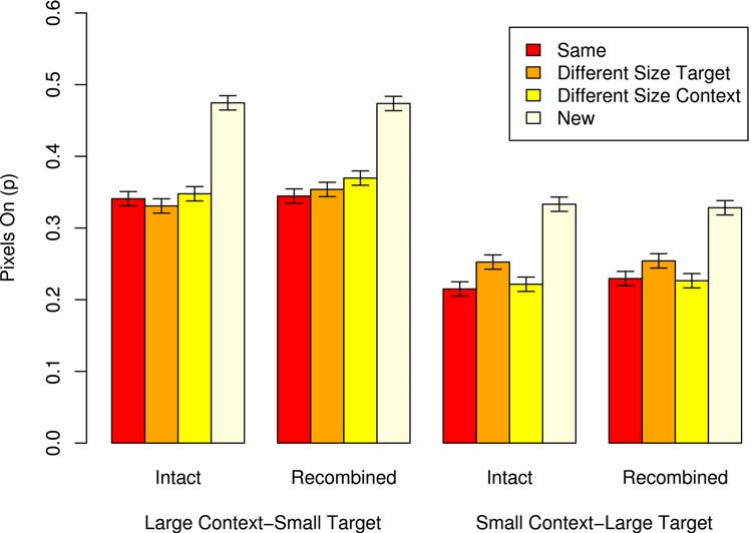
The mean proportion of pixels required for making ratings on the relative size rating test for photos of objects, as a function of targets' relative size at test (small, large), study/test context (intact, recombined), study/test relative size condition (same, different size target, different size context), and history (studied, non-studied). Error bars denote 95% within-subject confidence intervals.

Figure 2 shows the mean proportions of pixels required for making object size decisions in each experimental and control condition (error bars denote 95% within-subjects confidence intervals [44]). For non-studied objects, participants were faster to make size decisions when they were shown as relatively large (0.331 pixels corresponding to 6.62 s) than when they were shown as relatively small (0.474 pixels corresponding to 9.48 s). An ANOVA of performance on non-studied targets showed a significant effect of relative size, *F*(1,95) = 199.31, *MSe* = 0.010, η^2^ = .68.

The means in Figure 2 reveal priming: Overall, a smaller proportion of visible pixels was required for rating studied (0.290 corresponding to 5.8 s) than non-studied (0.402 corresponding to 8.04 s) targets in all experimental conditions [smallest *t*(95) = 4.18]. Because of the differences in baseline performance (i.e., performance on non-studied targets), priming scores were used for all further statistical analyses.

The critical question examined whether priming is influenced by the study-to-test changes in relative size. Figure 2 shows that study-to-test changes in relative size influenced priming but only when the target size changed between study and test and only for target objects displayed as large at test. Specifically, priming was larger when the target size was the same at study and test (0.109) than when it was different (0.078). In contrast, priming was not affected by study/test changes in context object size. For objects displayed small at test, priming was comparable in the same (0.133), different target size (0.132), and different context size (0.115) conditions. Finally, Figure 2 indicates that there was only a small influence due to study-to-test changes in context objects (i.e., intact/recombined pair manipulation).

These observations were confirmed by two sets of ANOVAs of the priming scores. First, an omnibus ANOVA of the priming scores that had target relative size at test (small, large), study/test relative size condition (same, different size target, different size context), and study/test context (intact, recombined) as within subject factors showed the following significant main effects and interactions: target relative size at test, *F*(1,95) = 7.09, *MSe* = 0.033, *η* ^2^ = .07; study/test context (intact, recombined), *F*(1,95) = 12.27, *MSe* = 0.003, *η* ^2^ = .11; study/test relative size condition, *F*(2,190) = 7.08, *MSe* = 0.004, *η* ^2^ = .07; interaction between target size at test and study/test relative size condition, *F*(2,190) = 16.30, *MSe* = 0.003, *η* ^2^ = .15. No other effects approached significance. Follow-up ANOVAs of the priming scores for targets displayed small at test with study/test relative size condition as a within-subject factor showed a significant main effect of study/test relative size condition, *F*(2,190) = 4.56, *MSe* = 0.004, *η* ^2^ = .05. Follow-up ANOVAs of the priming scores for targets displayed large at test with study/test relative size condition as within subject factor showed a significant main effect of study/test relative size condition, *F*(2,190) = 21.72, *MSe* = 0.003, *η* ^2^ = .19.

Second, planned ANOVAs of the priming scores focused on three contrasts: (1) priming for the intact pairs in the same size vs. different target size conditions; (2) priming for the intact pairs in the same size vs. different context size conditions; and (3) priming in the same relative size condition for the intact vs. recombined pairs. The first ANOVA of priming scores for the intact pairs with target test size and study/test relative size (same, different target size) as within subject factors showed a significant main effect of target test size, *F*(1, 95) = 11.08, *MSe* = 0.015, *η* ^2^ = .10, a significant main effect of study/test change in target size, *F*(1, 95) = 5.30, *MSe* = 0.004, *η* ^2^ = .05, and a significant interaction between target test size and study/test change in target size, *F*(1, 95) = 14.81, *MSe* = 0.004, *η* ^2^ = .13. A follow-up simple effects analysis showed that the main effect of study/test target size was significant for large test targets, *F*(1, 95) = 23.72, *MSe* = 0.003, *η* ^2^ = .20, but not for small test targets, *F*<1, *η* ^2^ = .008. The second ANOVA of priming scores for the intact pairs with target test size and study/test relative size (same, different context size) showed no significant effects [target test size, *F*(1, 95) = 1.89, *MSe* = 0.015, *η* ^2^ = .02; study/test relative size, *F*(1, 95) = 1.35, *MSe* = 0.004, *η* ^2^ = .01; interaction between target relative size at test and study/test relative size, *F*(1, 95) = 0.01, *MSe* = 0.003, *η* ^2^ = <.01]. The third ANOVA of the priming scores in the same relative size conditions with target test size and study/test context (intact/recombined) as within-subject factors showed a marginal effect of target relative size at test, *F*(1, 95) = 3.85, *MSe* = 0.014, *η* ^2^ = .04, p = .053. No other effects approached significance.

### Old/New Recognition

**Figure 3:**
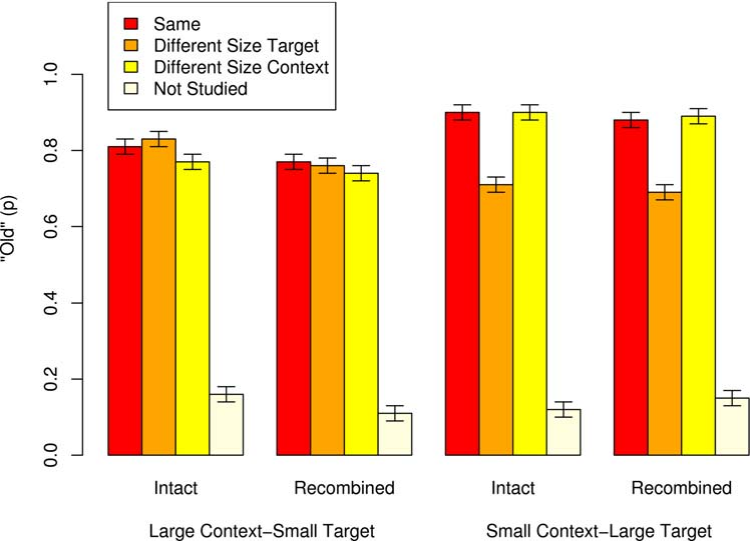
The proportion of “old” decisions (hits for old items and false alarms for new items) on old/new recognition test for photos of objects, as a function of targets' relative size at test (small, large), study/test context (intact, recombined), study/test relative size condition (same, different size target, different size context), and history (studied, non-studied). Error bars denote 95% within-subject confidence intervals.

Figure 3 shows the performance on the old/new recognition test in terms of correct decisions for studied items and false alarms for non-studied items (error bars denote 95% within-subjects confidence intervals [44]). For non-studied targets, performance shows some variability across experimental conditions (0.14 for small targets and 0.13 for large targets). For this reason, all statistical analyses were conducted on scores corrected for baseline performance by subtracting false alarms (incorrect decisions on non-studied targets) from hits (correct decisions on studied targets).

For studied targets, the corrected scores revealed that overall participants were more accurate on targets displayed in large size (0.71) than those displayed in small size (0.65). Study/test changes in relative size influenced accuracy only when targets were displayed in large test size and when target size changed between study and test; accuracy was higher for objects displayed in large test size when target size was the same (0.76) at study and test than when it was different (0.58). There were no or only minimal effects due to study-to-test changes in contexts (accuracy was 0.69 for intact pairs and 0.67 for recombined pairs).

These observations were confirmed by two sets of ANOVAs. First, an omnibus ANOVA that had target test size (small, large), study/test relative size condition (same, different size target, different size context), study/test context (intact, recombined) as within-subject factors showed the following significant main effects and interactions: target test size, *F*(1,63) = 27.92, *MSe* = .03, *η* ^2^ = .31; study/test relative size condition, *F*(2,126) = 30.55, *MSe* = .02, *η* ^2^ = .33; interaction between target test size and study/test relative size condition, *F*(2,126) = 47.62, *MSe* = .02, *η* ^2^ = .43. No other effects approached significance [study/test context (intact, recombined), *F*(1,63) = 2.48, *MSe* = .03, *p* = .12, *η* ^2^ = .04; target test size by study/test context interaction, *F*(1,63) = 0.11, *MSe* = 0.03, *p* = 0.75, *η* ^2^<.01; study/test context by study/test relative size condition, F(2, 126) = 0.19, *MSe* = 0.03, *p* = 0.83, *η* ^2^<.01; target test size by study/test context by study/test condition interaction, *F*(2, 126) = 0.40, *MSe* = 0.02, *p* = 0.67, *η* ^2^<.01]. Follow-up ANOVAs in the small target test size condition with study/test relative size condition as a within-subject factor did not approach significance, *F*(2,126) = 2.06, *MSe* = 0.02, *p* = 0.13, *η* ^2^ = 0.03. A follow-up ANOVA in the large target test size conditions with study/test relative size condition as a within-subject factor was significant, *F*(2,126) = 79.67, *MSe* = 0.02, *η* ^2^ = .56. Simple effect analyses revealed that performance in the same study/test condition differed from the different size target condition, *F*(1,63) = 77.93, *MSe* = 0.03, *η* ^2^ = 0.55, but not from the different context size condition, *F*(1,63) = 3.16, *MSe* = 0.01, *p* = 0.08, *η* ^2^ = 0.05.

## Discussion

The findings replicate and extend several results from previous research. On the implicit memory test, the relative size decisions took much longer for new (non-studied) objects displayed small versus large at test, consistent with previous research showing that small vs. large objects are more difficult to identify [1], our experience in identifying objects from small vs. large photos, and our experience in identifying objects afar when higher spatial frequencies are lost from the image [1], [34]. Moreover, there was substantial priming in all test conditions due to prior study. Important new findings showed that study-test changes in target object size influenced repetition priming but only for objects displayed large at test, confirming the prediction of the asymmetric priming effect derived from the limitation of the human visual system. Study-test changes in context object size did not affect the size of repetition priming suggesting that relative size changes *combined with* absolute size changes reduce priming rather than relative size changes in general. The possibility that size effects on priming are simply due to absolute size changes between study and test is eliminated by the results of previous research: study to test changes of object absolute size had no or only minimal effects on priming in both our own previous study using color photos of common objects [1] as well as in previous studies using primarily line-drawings of objects (see Table 1). In combination, these results suggest that object size affects priming at least when object size violates our pre-existing knowledge, when it is surprising, and when the study task focuses participants' attention on object size (see also [30]).

On the explicit old/new recognition test, the accuracy of making old/new decisions was higher for objects displayed large at test and accuracy was lowered by study-test changes in object size but only for objects tested as large, consistent with both predictions derived from the limitations of the human visual system as well as prior findings [1], [33], [34]. The intact/recombined manipulation did not affect old/new recognition accuracy perhaps due to the relatively weak associative processing during study conferred by the relative size rating only. Associative effects are typically only found following study tasks that require participants to associate the two items (a context and a target) together by stronger means, for example, by asking participants to create meaningful sentences combining the context target pairs. To illustrate, in Uttl et al. [26], participants saw a context first, rated its pleasantness, and when a target appeared, they were required to create a meaningful sentence connecting the context and the target in a meaningful way. With this strong associative manipulation, Uttl et al. [26] found strong intact/recombined effects on old/new recognition.

In combination, our findings highlight that the results previously obtained with line-drawings of objects, for example, the lack of asymmetric size effects on explicit memory tests, do not necessarily generalize to color photos of objects. This asymmetry of study/test size change effects on implicit and explicit memory is consistent with the predictions derived from drawing a parallel between spatial resolution of each image displayed on a computer monitor and the spatial resolving power of the human visual system for objects seen from afar, when high spatial frequencies of retinal images of objects seen from afar cannot be resolved [1], [34]. To illustrate, if a large test display includes 100 features or bits of information whereas a small test display includes only 25 features, the stored representations of large objects include as many as 100 features whereas the stored representations of small objects can include only 25 features. Thus, participants are most likely to find a match between information provided by large test displays and information included in study trial representations of large but not small objects, and consequently, their performance will be more affected when they are tested using large vs. small test displays. In contrast, information provided by small test displays is likely included in the study representations of both small and large objects, and therefore, participants' performance is unaffected by study/test size manipulations.

It could be argued that the parallel effects of relative size on implicit and explicit memory tests are due to participants engaging in explicit memory strategies on putatively implicit memory tests. However, several lines of independent evidence strongly argue against this possibility. First, knowing whether or not objects appeared during prior study was irrelevant to participants' performance on the task rating the relative size of context and target objects. Second, the experimental design – a large number of trials and a seven-day delay – made it difficult if not impossible for participants to remember the decisions they made one week earlier. Third, performance on these two implicit and explicit memory tests is differentially affected by various other object attributes such as absolute size [1], object orientation [4], and object color [5], demonstrating functional dissociations between these two tests. Finally, performance on the implicit and explicit memory tests in this study was statistically independent. Specifically, the average within-subject correlation (each based on 72 items) between priming on the phase 2 implicit task and old/new recognition performance over the same items was *r* = 0.01, no different from zero (*p* = 0.93). If implicit test performance results were due to participants attempting to intentionally retrieve their response from the previous phase, we would expect a significant positive correlation between the implicit and explicit memory test performance. However, a zero correlation is a strong evidence of divergent test validity; it indicates that implicit memory test performance was not influenced by explicit memory retrieval. In contrast, the average within-subject correlations between priming on the phase 2 implicit task and priming one week later (phase 3 implicit task) was *r* = 0.36, *p*<0.002. While each of these lines of evidence alone have been cited in the past as evidence that implicit test performance was not due to explicit memory retrieval, collectively, these four lines of evidence make it nearly impossible that participants engaged in explicit recollection on this implicit memory test and that such explicit recollection is responsible for the results obtained [45], [46].

The findings of size-specific priming effects with common objects parallels previous reports of priming effects specific to the color and spatial orientation in which objects were displayed for study and test [4], [5]. None of these effects can be readily accommodated by models that explain priming effects in terms of abstract structural representations (e.g., [2], [8], [9], [47]). For example, Biederman's recognition-by-components model does not include any provision for coding size, colour, or other episodic information. According to this model, it is assumed that the structural representation of the geons alone is the only information necessary to identify and remember objects; results from our lab and others though suggest a necessary revision to this tenet. Similar to Biederman's model, Tulving and Schacter [9] have also postulated that object identification and priming is mediated by representations of structural properties. Their model differs, however, in that they suggest that the structural description system is closely linked with the episodic memory system, and that this system can code objects in terms of specific properties (e.g., color, spatial orientation, size, context) that are unique to each occurrence. Therefore, hybrid models such as this one can accommodate our findings of size-specific priming effects by assuming that the identification of an object recruits both its structural descriptions as well as its episodic memory representations.

Instead of highlighting the contributions of potentially different representation systems (e.g., structural, episodic), other researchers have explained repetition priming effects in terms of instance representations [4], [11], [22]–[26], [28], [29]. According to these views, each encounter with a stimulus engages a unique set of sensory and perceptual processes, and as a consequence, this same set of processes can be carried out more fluently in the future. The enduring consequence of processing an item is regarded as its episodic memory representation. It is assumed that when required to identify a familiar object, both new and preexisting representations of the object are recruited for its identification and influence how it is perceived, interpreted and encoded. This new episodic representation will then influence subsequent identification less to the extent that an object already has many pre-existing representations in memory. By these views, identification is a dynamic process and the influence of various object attributes such as size or perceived size on identification and priming depends not only on a match between attributes present at study and test but on a number of factors, including participants' familiarity with a specific view of objects including their perceived size, requirements of study and test tasks that focus attention either towards or away from the processing of a specific attribute (e.g., size, color, orientation), and cues provided at test (which may include or exclude the specific attribute).

Our findings of substantial size-specific influences on priming with common objects under some but not other conditions are consistent with instance views of object perception and priming but inconsistent with pure structural description views. These size-specific influences complement our previous research showing orientation and color-specific effects on object priming. More importantly, this combination of findings strengthens the claim that priming effects with common objects are similar to priming effects with written and spoken words, with names, faces, et cetera. They suggest that we should abandon theoretical accounts that are unique to one kind of material in favor of accounts that cover findings across various materials including words, objects, and faces.

Finally, our findings have forensic implications. They show that participants' ability to recognize previously seen objects as previously seen is substantially affected by the size (or distance) in which the object was initially encountered. If an object was seen small or from afar, eyewitnesses are far less likely to correctly identify the object as being present in the crime scene than if it was seen large or from nearby. In turn, our findings support the proposal for using object arrays or lineups for cross-examining eyewitnesses about their ability to correctly identify crime scene objects [1], [48].

## References

[1] Uttl B, Graf P, Siegenthaler AL (2007). Influence of object size on baseline identification, priming, and explicit memory.. Scandinavian Journal of Psychology.

[2] Biederman I (1987). Recognition-by-components: a theory of human image understanding.. Psychological Review.

[3] Marr D, Nishihara HK (1978). Representation and recognition of the spatial organization of three-dimensional shapes.. Proc R Soc Lond, B, Biol Sci.

[4] Uttl B, Graf P (1996). Object orientation information in semantic and episodic memory.. Canadian Journal of Experimental Psychology.

[5] Uttl B, Graf P, Santacruz P (2006). Object color affects identification and repetition priming.. Scandinavian Journal of Psychology.

[6] Tarr MJ (n.d.). Rotating objects to recognize them: A case study on the role of viewpoint dependency in the recognition of three-dimensional objects.. Psychonomic Bulletin & Review.

[7] Tarr MJ, Pinker S (1989). Mental rotation and orientation-dependence in shape recognition.. Cognitive Psychology.

[8] Schacter DL, Schacter DL, Tulving E (1994). Priming and multiple memory systems: Perceptual mechanisms of implicit memory.. Memory systems 1994.

[9] Tulving E, Schacter DL (1990). Priming and human memory systems.. Science.

[10] Roediger HL, Blaxton TA (1987). Effects of varying modality, surface features, and retention interval on priming in word-fragment completion.. Memory & Cognition.

[11] Graf P, Ryan L (1990). Transfer-appropriate processing for implicit and explicit memory.. Journal of Experimental Psychology: Learning, Memory, and Cognition.

[12] Jacoby LL, Hayman CA (1987). Specific visual transfer in word identification.. Journal of Experimental Psychology: Learning, Memory, and Cognition.

[13] Kolers PA (1973). Remembering operations.. Memory & Cognition.

[14] Kolers PA (1976). Reading a year later.. Journal of Experimental Psychology: Human Learning and Memory.

[15] Rudnicky AI, Kolers PA (1984). Size and case of type as stimuli in reading. Journal of Experimental Psychology.. Human Perception and Performance.

[16] Masson ME (1986). Identification of typographically transformed words: Instance-based skill acquisition.. Journal of Experimental Psychology: Learning, Memory, and Cognition.

[17] Graf P, Shimamura AP, Squire LR (1985). Priming across modalities and priming across category levels: extending the domain of preserved function in amnesia.. Journal of Experimental Psychology. Learning, Memory, and Cognition.

[18] Jacoby LL, Dallas M (1981). On the relationship between autobiographical memory and perceptual learning. Journal of Experimental Psychology.. General.

[19] Church BA, Schacter DL (1994). Perceptual specificity of auditory priming: implicit memory for voice intonation and fundamental frequency.. Journal of Experimental Psychology. Learning, Memory, and Cognition.

[20] Craik FIM, Kirsner K (1974). The effect of speaker's voice on word recognition.. The Quarterly Journal of Experimental Psychology.

[21] Schacter DL, Church BA (1992). Auditory priming: Implicit and explicit memory for words and voices.. Journal of Experimental Psychology: Learning, Memory, and Cognition.

[22] Craik FIM, Bowers JS, Marsolek CJ (2003). Commentary.. Rethinking implicit memory.

[23] Feustel TC, Shiffrin RM, Salasoo A (1983). Episodic and lexical contributions to the repetition effect in word identification. Journal of Experimental Psychology.. General.

[24] Logan GD (1988). Toward an instance theory of automatization.. Psychological Review.

[25] Whittlesea B, Bowers JS, Marsolek CJ (2003). On the construction of behavior and subjective experience: the production and evaluation of performance.. Rethinking implicit memory.

[26] Uttl B, Graf P, Cosentino S, Bowers JS, Marsolek CJ (2003). Implicit memory for new associations: Types of conceptual representations.. Rethinking implicit memory.

[27] Thoma V, Davidoff J, Hummel JE (2007). Priming of plane-rotated objects depends on attention and view familiarity.. Visual Cognition.

[28] Mandler G (1980). Recognizing: The judgment of previous occurrence.. Psychological Review.

[29] Rueckl JG, Bowers JS, Marsolek CJ (2003). A connectionist perspective on repetition priming.. Rethinking implicit memory.

[30] Srinivas K (1996). Size and reflection effects in priming: a test of transfer-appropriate processing.. Memory & Cognition.

[31] Biederman I, Cooper EE (1992). Size invariance in visual object priming. Journal of Experimental Psychology.. Human Perception and Performance.

[32] Jolicoeur P (1987). A size-congruency effect in memory for visual shape.. Memory & Cognition.

[33] Kolers PA, Duchnicky RL, Sundstroem G (1985). Size in the visual processing of faces and words.. Journal of Experimental Psychology: Human Perception and Performance.

[34] Loftus GR, Harley EM (2005). Why is it easier to identify someone close than far away?. Psychonomic Bulletin & Review.

[35] Campbell FW, Robson JG (1968). Application of Fourier analysis to the visibility of gratings.. J Physiol (Lond.).

[36] Thibos LN, Cheney FE, Walsh DJ (1987). Retinal limits to the detection and resolution of gratings.. Journal of the Optical Society of America. A, Optics and Image Science.

[37] Osterberg GA (1935). Topography of the layer of rods and cones in the human retina.. Acta Ophthalmologica.

[38] Snodgrass JG, Feenan K (1990). Priming effects in picture fragment completion: Support for the perceptual closure hypothesis.. Journal of Experimental Psychology: General.

[39] Graf P, Schacter DL (1985). Implicit and explicit memory for new associations in normal and amnesic subjects.. Journal of Experimental Psychology. Learning, Memory, and Cognition.

[40] Graf P, Schacter DL (1987). Selective effects of interference on implicit and explicit memory for new associations.. Journal of Experimental Psychology: Learning, Memory, and Cognition.

[41] Graf P, Schacter DL (1989). Unitization and grouping mediate dissociations in memory for new associations.. Journal of Experimental Psychology: Learning, Memory, and Cognition.

[42] Schacter DL, Graf P (1986). Effects of elaborative processing on implicit and explicit memory for new associations.. Journal of Experimental Psychology: Learning, Memory, and Cognition.

[43] Schacter DL, Graf P (1989). Modality specificity of implicit memory for new associations.. Journal of Experimental Psychology. Learning, Memory, and Cognition.

[44] Masson MEJ, Loftus GR (2003). Using confidence intervals for graphically based data interpretation.. Canadian Journal of Experimental Psychology.

[45] Poldrack R (1996). On testing for stochastic dissociations.. Psychonomic Bulletin & Review.

[46] Tulving E, Hayman CAG (1993). Stochastic independence in the recognition/identification paradigm.. European Journal of Cognitive Psychology.

[47] Marr D (1982). Vision.

[48] Steele LJ (1998). Physical evidence lineups: An argument which deserves exploration.. Criminal Law Bulletin.

[49] Cave CB, Squire LR (1992). Intact and long-lasting repetition priming in amnesia.. Journal of Experimental Psychology. Learning, Memory, and Cognition.

[50] Fiser J, Biederman I (1995). Size invariance in visual object priming of gray-scale images.. Perception.

[51] Zimmer HD (1995). Size and orientation of objects in explicit and implicit memory: a reversal of the dissociation between perceptual similarity and type of test.. Psychological Research.

[52] Seamon JG, Ganor-Stern D, Crowley MJ, Wilson SM, Weber WJ (1997). A mere exposure effect for transformed three-dimensional objects: effects of reflection, size, or color changes on affect and recognition.. Memory & Cognition.

[53] Furmanski CS, Engel SA (2000). Perceptual learning in object recognition: object specificity and size invariance.. Vision Res.

[54] Stankiewicz BJ, Hummel JE (2002). Automatic priming for translation- and scale-invariant representations of object shape.. Visual Cognition.

[55] Ryan CS, Hemmes NS, Brown BL (2003). The effect of chromaticity varies with object identification response: speeded naming versus recognition.. The Psychological Record.

